# Refractive change following pseudophakic vitrectomy

**DOI:** 10.1186/1471-2415-8-19

**Published:** 2008-10-13

**Authors:** Sinead Byrne, James Ng, Anthony Hildreth, Jean-Pierre Danjoux, David HW Steel

**Affiliations:** 1 Sunderland Eye Infirmary, Queen Alexandra Road, Sunderland, Tyne and Wear, UK; 2 Clinical Trials Unit, Sunderland Royal Hospital, Kayll Road, Sunderland, Tyne and Wear, UK

## Abstract

**Background:**

To assess the occurrence and magnitude of refractive change in pseudophakic eyes undergoing 20 gauge pars plana vitrectomy without scleral buckling and to investigate possible aetiological factors.

**Methods:**

Retrospective case note review of 87 pseudophakic eyes undergoing 20 gauge pars plana vitrectomy for a variety of vitreo-retinal conditions over a three-year period. Anterior chamber depth (ACD) was measured before and after vitrectomy surgery in 32 eyes. Forty-three pseudophakic fellow eyes were used as controls.

**Results:**

Eighty-seven eyes (84 patients) were included in the study. Mean spherical equivalent refraction prior to vitrectomy was -0.20 dioptres, which changed to a mean of -0.65 dioptres postoperatively (standard deviation of refractive change 0.59, range-2.13 to 0.75 dioptres) (p < 0.001). Sixty-one of the 87(70%) eyes experienced a myopic shift and 45(52%) eyes had a myopic shift of -0.5 dioptres or more. Mean fellow eye refraction was -0.19 dioptres preoperatively and -0.17 dioptres postoperatively (p = 0.14)(n = 37)

Mean ACD preoperatively was 3.29 mm and postoperatively 3.27 mm (p = 0.53) (n = 32) and there was no significant change in ACD with tamponade use. Regression analysis revealed no statistically significant association between changes in anterior chamber depth, as well as a wide variety of other pre-, intra and postoperative factors examined, and the refractive change observed.

**Conclusion:**

Significant refractive changes occur in some pseudophakic patients undergoing 20 g pars plana vitrectomy. The mean change observed was a small myopic shift but the range was large. The aetiology of the refractive change is uncertain.

## Background

The refractive index of the vitreous, being 99% water, is virtually identical to that of aqueous, and hence vitrectomy, without scleral buckling, in pseudophakic eyes is not thought conventionally to effect refraction. Corneal topographic changes have been noted after vitrectomy, but corneal shape returns to pre operative levels within three months [1,2]. We recently had experience of a pseudophakic patient who had a clinically noticeable myopic shift in his refraction after 20 gauge vitrectomy for macular on retinal detachment (See Table 1). We decided therefore to investigate refractive status pre and post vitrectomy in a series of pseudophakic patients undergoing vitrectomy and examine any factors that might explain and predict refractive change

**Table 1:** Case summary

A 32-year-old man underwent clear lens extraction with phacoemulsification and intraocular lens implant for the correction of high myopia (refraction right eye -15 dioptres, axial length 30.12 mm) after problems with contact lens intolerance. Postoperatively he was pleased with the result and achieved acuity in his right eye of 6/18 unaided which improved to 6/6 with a correction of +1/+1 at 90°. He maintained stable vision but two years following surgery he presented with a two-day history of a visual floaters and an infero-nasal field defect and was found to have a supero-temporal retinal detachment with the macula still just attached. Vitrectomy surgery was carried out the same day using self-sealing 20 g sclerostomies with cryotherapy to a single superior horseshoe tear and SF6 gas tamponade. On postoperative review his retina was attached and he was pleased with his unaided vision, which had improved to 6/9. Three months following surgery his vision was stable with a refractive correction of 0/+1 at 90° which remained unchanged over a follow up period of 24 months.

## Methods

We reviewed the case notes of pseudophakic patients undergoing three-port pars plana vitrectomy for a variety of vitreo-retinal conditions over a three-year period.

All the vitrectomy procedures were carried out by one surgeon using a 20-gauge three-port pars plana technique with self-sealing scleral tunnels created perpendicular to the limbus. Patients requiring silicone oil tamponade, scleral buckling surgery or intraoperative IOL manipulations were excluded.

The following criteria were essential for inclusion:

1) An inter-operative period of greater than four months between phacoemulsification and vitrectomy to allow refractive stabilisation.

2) Availability of refractive data obtained pre vitrectomy at least three months post cataract surgery and within six months prior to vitrectomy surgery.

3) Availability of data from a refraction performed three to four months post vitrectomy.

4) Details of Intraocular lens (IOL) power and type documented at the time of the original cataract extraction.

The following data was recorded from the notes: Date of cataract surgery, type and power of intra ocular lens implant used, axial length prior to vitrectomy, indication for vitrectomy, time between vitrectomy and cataract extraction, vitreo-retinal procedure undertaken, including type of tamponade agent used, refraction before and after vitrectomy and the occurrence of any intra or postoperative problems.

Anterior chamber depth (ACD) (in this study taken as the distance between the anterior IOL surface and the corneal endothelium) was measured in 32 patients one day prior to, and three months following vitrectomy using the Zeiss IOL Master. Measurements were taken with the patient seated in an upright position under standardised lighting conditions and an average of 10 readings was used. Control refractive data was obtained on 43 pseudophakic fellow eyes.

Statistical analysis was carried out to assess the degree of refractive change from pre to post vitrectomy, and to determine any correlation of refractive change observed and a variety of pre, intra and post operative factors.

### Statistical analysis

Statistical analysis of the data was undertaken using SPSS Version 15.0 and S-Plus professional Version 6.2. Change in refraction was analysed via a linear regression model, the fit of which was assessed via examining residuals. Subsequent analysis of the remaining variables was via a general linear model.

## Results

Eighty-seven eyes of 84 patients were included. Forty-five were female and 39 male. The mean age was 65 years old with a range of 38–84 years.

Indications for vitrectomy surgery were macula on retinal detachment in 17(19%) eyes, macular off retinal detachment in 15(17%), macular pucker in 15(17%), macular hole in 15(17%), proliferative diabetic retinopathy in 14(16%), and miscellaneous in 11(13%). In the eyes with retinal detachment, retinal breaks were in the superior retina in 17, inferior in 5 and mixed inferior and superior quadrants in 10.

Tamponade with air or SF6 was used in 24(28%) and C3F8 in 34(39%). No tamponade agent was used in the other patients.

The mean time from cataract surgery to vitrectomy surgery was 39 months (median 28 months, range 4–149 months).

Four groups of intraocular lens type were noted: 53(61%) were folding three piece acrylic IOLs, 10(11%) were folding acrylic one piece lenses, 6(7%) were silicone plate lenses and 18(21%) were rigid polymethylmethacrylate (PMMA) lenses – 6 of these were following extracapsular cataract surgery. All other eyes in the study had had small incision phacoemulsification surgery carried out. None of the eyes had corneal sutures present at the time of vitrectomy. Three eyes in the folding three piece acrylic IOL group and two from the rigid PMMA group had had documented posterior capsule rupture with sulcus fixated IOL implantation. All other eyes were documented as the IOL having been inserted in the capsular bag at the time of cataract surgery. There were no eyes with manifestly unstable IOL position in the study.

IOL power ranged from 2 to 33 dioptres (mean 20.41 dioptres). Axial length ranged from 19.50 mm to 34.01 mm (mean 23.71 mm).

Five eyes experienced a rise in intraocular pressure immediately postoperatively to greater than 35 mm hg. All were successfully treated and pressure was controlled within 24 hours. Thirty-two eyes had a surgical capsulotomy performed during the vitrectomy procedure and 12 eyes had had a prior YAG laser capsulotomy.

Refraction prior to vitrectomy ranged from -2.75 to +2.63 dioptres spherical equivalent with a mean of -0.20 dioptres (standard deviation (SD) 1.15 dioptres). Postoperatively this changed to a mean spherical equivalent of -0.65 dioptres representing a significant change of -0.45 dioptres (SD 0.59, range -2.13 to 0.75 dioptres) (p < 0.001). Sixty-one of the 87(70%) eyes experienced a myopic shift and 45(52%) eyes had a myopic shift of -0.5 dioptres or more. The mean change in astigmatism as assessed by the pre and postoperative refraction was 0.28 dioptres (SD 0.63) with a normalised change of 8.3 degrees with the rule.

Mean ACD preoperatively was 3.29 mm (SD 0.79) and postoperatively 3.27 mm (SD 0.71) (Paired t test, n = 32, p = 0.53)

We compared ACD change between categories of tamponade agent used (no tamonade, SF6 and C3F8) via an analysis of variance model (ANOVA) after confirming that that ACD change was normally distributed within each category. There was no statistically significant difference between groups for depth change. (ANOVA, n = 87, p = 0.66)

Mean fellow eye refraction was -0.19 dioptres preoperatively and -0.17 dioptres postoperatively representing a change of 0.02 dioptres (Wilcoxon matched pairs test, n = 43, p = 0.14). This was significantly different from the change of -0.45 dioptres in the eyes undergoing vitrectomy (Mann-Whitney test, p < 0.0001)

### Regression model

After assessing the appropriateness of the linear model via preliminary analysis, the following variables were entered into a stepwise linear regression model: pre-operative refraction, age, change in astigmatism, gas tamponade use, diagnosis category, IOL type, IOL power, presence of capsulotomy, length of time from previous cataract surgery, raised intraocular pressure post-operatively, and axial length. Also entered into the model were pre-operative ACD and change in AC depth (n = 32). A regression model with pairwise exclusion failed where missing values remained for the ACD variables, therefore these cells were replaced with mean values, the ensuing model resulting in an almost identical model to that obtained when excluding these two variables. Eleven of the variables were excluded from the final model, the two remaining variables being pre-operative refraction and gas code. The R squared for the final model was 0.08, indicating that only 8.2% of the variance in refraction change was due to pre-operative refraction and gas tamponade (n = 87). Standardised beta coefficients for the final model were -0.095 (p = 0.085) for pre-operative refraction and -0.17 (p = 0.026) for gas code.

### General linear model

Analysis of covariance was used to test the effect on change in refraction of gas tamponade, entering pre-operative refraction as a covariate. Although neither variable showed a statistically significant value at the 5% level, (tamponade use p = 0.084, pre-operative refraction p = 0.09), these results tended to corroborate the regression model suggesting that that they may be mildly prognostic. Adjusted mean (95% confidence interval) changes in refraction were *no gas *-0.28 (-0.49 to -0.06), *SF6 gas *-0.47 (-0.70 to -0.23), *C3F8 *gas -0.61 (-0.81 to -0.41). P-values failed to reach significance at the 5% level, however the difference between *no gas *and *C3F8 gas *was significant to p = 0.08 (Table 2).

**Table 2:** Mean differences in refractive change with tamponade use

Comparison	Mean Difference	Std. Error	p-value
None – SF6	0.19	0.16	0.72
None – C3F8	0.33	0.15	0.08
SF6 – C3F8	0.14	0.16	1.00

**Figure 1:**
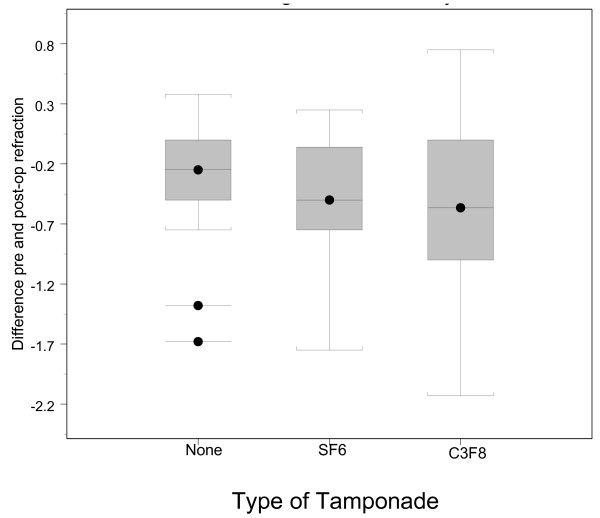
Difference in pre and post operative refractions in each group.

Figure 1 shows the distribution of change in refraction by tamponade use (see additional files). Thirty four patients received C3F8, 24 received SF6, and 29 did not receive a tamponade.

## Discussion

We noticed a small myopic shift in refraction of -0.45 dioptres four months following vitrectomy in this series of pseudophakic patients. The degree of refractive change was variable ranging from +0.75 to -2.13 dioptres but 52% of eyes experienced a clinically noticeable myopic shift of -0.5 dioptres or more. The study was limited by its retrospective nature and the large number of surgical variables however other authors have also incidentally recorded similar changes in studies evaluating the success of vitrectomy in pseudophacic retinal detachment. Sharma in a study comparing scleral buckling surgery to vitrectomy in the treatment of pseudophakic retinal detachment, found a mean change of -0.84 dioptres with vitrectomy surgery alone [3]. Campo et al found a -0.15 dioptre change after vitrectomy surgery again in a study of vitrectomy for pseudophakic retinal detachment [4]. The exact time point when refractive change was assessed was not clear in the latter two papers.

There are a number of possible explanations for the myopic shift that we found. We hypothesised that vitrectomy, especially if combined with gas tamponade, could result in an anterior movement of the IOL position, resulting in a myopic shift especially in cases of a sulcus fixated IOL, flexible IOL or in cases with a large capsulorhexis. This has been proposed as a possible explanation for the small myopic shift seen after combined phacovitrectomy surgery with gas tamponade [5] and by Sharma et al. for the myopic shift they observed following vitrectomy for retinal detachment [3]. ACD has been shown to vary in the first three months post-operatively following cataract surgery with refractive change [6], hence we specifically excluded patients having vitrectomy surgery within four months of cataract surgery.

We assessed ACD pre and postoperatively in 32 patients, however we did not find any significant change in ACD and there was no association between ACD pre-operatively and the change in postoperative refraction. We also found no association between IOL type, time between cataract surgery and vitrectomy surgery, IOL power or axial length and refractive change. If IOL position change was causing the myopic shift, then an association between these may have been expected. Importantly there was also no significant difference in ACD change and the use of tamponade agents. There were however no patients with a manifestly unstable IOL position in the study and the results may have been different if patients such as they had been included.

Vitreous has a refractive index of 1.336, which is identical to that of aqueous, and so we do not think that vitreous removal resulted in any significant refractive change.

Posterior capsulotomies were performed intra operatively during vitrectomy in 32 of the 87 eyes and twelve eyes had pre-existing laser capsulotomies. However no myopic shift has been associated with capsulotomy in non-vitrectomised eyes [7,8] and furthermore, there was no correlation between the occurrence of capsulotomy and the myopic shift we observed.

Three-port pars plana vitrectomy has been associated with a steepening of the central cornea in the immediate postoperative period as summarised by Randlemann [9]. Although our study was weakened by the absence of keratometric and longitudinal refractive data we found no clinically significant astigmatic changes as assessed by pre and postoperative refractions three months following pseudophakic vitrectomy. We also found no association of astigmatic change and the mean refractive change following vitrectomy. Studies looking at corneal astigmatism following vitrectomy surgery have found that any astigmatic changes associated with surgery resolve by three months [1,2] which is why we did not assess refraction any earlier than this time point. We excluded patients within four months of cataract surgery to avoid changing refraction confounding the results. Similarly we only included patients if they had had a refraction carried out within 6 months of vitrectomy for the same reason. Modern small incision cataract surgery is associated with rapid refractive stabilisation and by four months refraction is stable [10,11]. There were six patients in the study who had had large incision extracapsular cataract surgery carried out but these had all been carried out at least seven years prior to the vitrectomy. Significant new refractive change was unlikely to have occurred between refraction prior to vitrectomy (a maximum time of six months) and the vitrectomy procedure. The stability of refractive status in the eyes studied prior to vitrectomy is illustrated by the stability of the fellow eyes used as controls. We did not find an association between either the length of time from cataract surgery to vitrectomy surgery nor the IOL type (both surrogate markers of different types of cataract surgery and incision) and the change in refraction with vitrectomy. Corneal topography was not carried out and we cannot exclude changes in corneal steepening, perhaps associated with the 20 g sclerostomies used. It should be noted however that our findings may have been very different if eyes that had had more recent cataract surgery especially with larger incisions had been included.

A possible explanation for the myopic shift seen during phacovitrectomy surgery [5,12] is that a falsely short axial length is measured when macular thickening is present. Potentially this could also account for a refractive change in pseudophakic eyes with macular pathology after correction of the abnormality with vitrectomy surgery. However, this is not the explanation in our cases as in all but seven cases, the pre-operative refraction predated the evolution of the retinal pathology corrected by the vitrectomy surgery.

It is possible that a true increase in axial length after vitrectomy could have caused the myopic shift observed, possibly with post-operative stretching of sclerostomies associated with increased intra ocular pressure. Unfortunately we did not measure axial length post-operatively and therefore we cannot provide any data on this. Braztikos et al found a small but significant increase in axial length of 0.1 mm in a series of eyes undergoing vitrectomy without scleral buckling as compared to a study group undergoing scleral buckling surgery alone [13]. A change in axial length of this order could produce the refractive changes we have seen [14]. Jeoung et al. recently reported the results of a prospective study investigating factors influencing the refractive outcome of patients undergoing combined phacovitrectomy. Eyes with axial lengths greater than 24.5 mm experienced a myopic shift in achieved refraction from predicted of the order of -0.40 dioptres. They observed an apparent increase in axial length in these eyes and postulated that this may be a true increase in axial length associated with scleral stretching or thinning [15]. Interestingly we found some association, albeit not statistically significant (p = 0.08), between the use of C3F8 gas compared to no tamponade use and the occurrence of a myopic shift in refraction. Plausibly C3F8 use could result in forces favouring globe expansion especially in association with the 20 g self-sealing incisions we used. In effect the self-sealing 20 g sclerostomies may have acted as "scleral expansion surgery" for "the correction of hypermetropia". It would be interesting to study refractive change in patients undergoing conventionally sutured 20 g surgery and narrow gauge vitrectomy surgery in a prospective randomised study with measurement of axial length, keratometry and anterior chamber depth pre and postoperatively.

## Conclusion

A clinically noticeable persistent refractive changes occur in some pseudophakic patients undergoing 20 g pars plana vitrectomy. It is important for clinicians to be aware of this possibility and be able to counsel patients appropriately. The mean change we observed was a small myopic shift but the range was large. We were unable to clearly define the aetiology of the refractive change. Further prospective studies with measurement of axial length and keratometry pre and postoperatively are needed.

## Competing interests

The authors declare that they have no competing interests.

## Authors' contributions

SB carried out the literature review, data collection and helped draft the manuscript. JN assisted in data collection and literature review. AH performed the statistical analysis. JPD carried out the astigmatism analysis. DS conceived of the study, participated in its design and drafted the final manuscript.

## Pre-publication history

The pre-publication history for this paper can be accessed here:



## References

[B1] Cisiecki S, Nawrocki J (2005). [Vector analysis of surgically induced astigmatism after combined operation of phacoemulsification, intraocular lens implantation and pars plana vitrectomy]. Klin Oczna.

[B2] Eckert T, Eckardt C (1996). [Outcome of corneal astigmatism after pars plana vitrectomy with or without simultaneous cataract extraction]. Ophthalmologe.

[B3] Sharma YR, Karunanithi S, Azad RV (2005). Functional and anatomic outcome of sceral buckling versus primary vitrectomy in pseudophacic retinal detachment. Acta Ophthalmol Scand.

[B4] Campo RV, Sipperly JO, Scott R (1999). Pars plana vitrectomy without scleral buckling for pseudophacic retinal detachment. Ophthalmology.

[B5] Suzuki Y, Sakuraba T, Mizutani H, Matsuhashi H, Nakazawa M (2000). Postoperative refractive error after simultaneous vitrectomy and cataract surgery. Ophthalmic Surg Lasers.

[B6] Cekic O, Batman C, Totan Y, Emre I, Zilelioglu O (1998). Changes in anterior chamber depth and intraocular pressure after phacoemulsification and posterior chamber intraocular lens implantation. Ophthalmic Surgery and Lasers.

[B7] Hu CY, Woung LC, Wang MC, Jian JH (2000). Influence of laser posterior capsulotomy on anterior chamber depth, refraction, and intraocular pressure. J Cataract Refract Surg.

[B8] Findl O, Drexler W, Menapace R (1999). Changes in intraocular lens position after neodynium:Yag capsulotomy. J Cataract Refract Surg.

[B9] Randleman JB, Hewitt SM, Stulting RD (2004). Refractive changes after posterior segment surgery. Ophthalmol Clin North Am.

[B10] Zheng L, Merriam JC, Zaider M (1997). Astigmatism and visual recovery after'large incision' extracapsular cataract surgery and 'small' incisions for phacoemulsification. Trans Am Ophthalmol Soc.

[B11] Oshika T, Tsuboi S (1995). Astigmatic and refractive stabilisation after cataract surgery. Ophthalmic Surgery.

[B12] Shioya M, Ogino N, Shinjo U (1997). Change in postoperative refractive error when vitrectomy is added to intraocular lens implantation. J Cataract Refract Surg.

[B13] Brazitikos P, Androudi S, Christen W, Stangos N (2005). Primary pars plana vitrectomy versus scleral buckle surgery for the treatment of pseudophacic retinal detachment. Retina.

[B14] McEwan JR, Massengill RK, Friedel SD (1990). Effect of keratometer and axial length measurement errors on primary implant power calculations. J Cataract Refract Surg.

[B15] Jeoung JW, Chung H, Yu HG (2007). Factors influencing refractive outcomes after combined phacoemulsification and pars plana vitrectomy. J Cataract Refract Surg.

